# IGF-Binding Proteins in Type-1 Diabetes Are More Severely Altered in the Presence of Complications

**DOI:** 10.3389/fendo.2016.00002

**Published:** 2016-01-29

**Authors:** Ashok Sharma, Sharad Purohit, Shruti Sharma, Shan Bai, Wenbo Zhi, Sithara Raju Ponny, Diane Hopkins, Leigh Steed, Bruce Bode, Stephen W. Anderson, Jin-Xiong She

**Affiliations:** ^1^Center for Biotechnology and Genomic Medicine, Augusta University, Augusta, GA, USA; ^2^Department of Biostatistics and Epidemiology, Augusta University, Augusta, GA, USA; ^3^Department of Pathology, Augusta University, Augusta, GA, USA; ^4^Pediatric Endocrine Associates, Atlanta, GA, USA

**Keywords:** IGF-1, insulin-like growth factor 1, IGF-binding proteins, diabetes mellitus, type 1, diabetic complications, IGFBP

## Abstract

**Aims:**

Reduced levels of free and total insulin-like growth factor 1 (IGF-I) have been observed in type-1 diabetes (T1D) patients. The bioavailability of IGF-I from the circulation to the target cells is controlled by multifunctional IGF-binding proteins (IGFBPs). The aim of this study was to profile serum IGFBPs in T1D and its complications.

**Design:**

We measured the IGFBP levels in 3662 patient serum samples from our ongoing Phenome and Genome of Diabetes Autoimmunity (PAGODA) study. IGFBP levels of four different groups of T1D patients (with 0, 1, 2, and ≥3 complications) were compared with healthy controls.

**Results:**

Three serum IGFBPs (IGFBP-1, -2, and -6) are significantly higher in T1D patients, and these alterations are greater in the presence of diabetic complications. IGFBP-3 is lower in patients with diabetic complications. Analyses using quintiles revealed that risk of T1D complications increases with increasing concentrations of IGFBP-2 (fifth quintile ORs: 18–60, *p* < 10^−26^), IGFBP-1 (fifth quintile ORs: 8–20, *p* < 10^−15^), and IGFBP-6 (fifth quintile ORs: 3–148, *p* < 10^−3^). IGFBP-3 has a negative association with T1D complications (fifth quintile ORs: 0.12–0.25, *p* < 10^−5^).

**Conclusion:**

We found that elevated serum levels of IGFBP-1, -2, and -6 were associated with T1D, and its complications and IGFBP-3 level was found to be decreased in T1D with complications. Given the known role of these IGFBPs, the overall impact of these alterations suggests a negative effect on IGF signaling.

## Introduction

Type-1 diabetes (T1D) patients have reduced levels of circulating free and total IGF-I with alterations in the other components of the insulin-like growth factor (IGF)-axis (1–6). IGF-I exerts anti-inflammatory and pro-survival effects on the vasculature, resulting in reduced vascular oxidant stress, apoptosis, and inflammatory signaling (7). A decrease in IGF-I activity has also been shown to promote cerebromicrovascular dysfunction (8), accelerate endothelial apoptosis, and reduce the regenerative capacity of endothelium (9). The bioavailability of IGF-I from the circulation to the target cells is controlled by multifunctional IGF-binding proteins (IGFBP1–6) (5). In serum, about 98% of IGFs are always bound to one of the IGFBPs. In addition to functioning as simple carrier proteins, IGFBPs regulate the endocrine actions of IGFs by controlling the IGF availability for IGF receptors, whereas, locally produced IGFBPs act as autocrine/paracrine regulators of IGF action (10, 11). Besides, several IGFBPs also have IGF-independent actions, modulating numerous processes in the extracellular environment and inside the cell (10, 12–15). Interestingly, IGFBPs have been shown to possess anti-inflammatory function independent of IGF. In human endothelial cells, IGFBPs can inhibit NF-κB activity using their own receptors and subsequently suppress monocyte adhesion to endothelial cells (16).

Several studies have indicated alterations in IGFBPs in T1D patients (1, 17–19); however, these studies have used relatively smaller sample sets. Due to large inter-individual variation in levels of the IGFBPs, larger sample sizes are needed to draw definite conclusions. Furthermore, the relationships of IGFBPs with accelerated micro-and macro-vascular complications are relatively unexplored. We measured the IGFBP-1, -2, -3, -6, and -7 levels in 3662 serum samples from our ongoing Phenome and Genome of Diabetes Autoimmunity (PAGODA)/Prospective Assessment in Newborns for Diabetes Autoimmunity (PANDA) study (20). We found that circulating levels of IGFBP-1, IGFBP-2, and IGFBP-6 are moderately higher in T1D patients. In the presence of diabetic complications, circulating levels of IGFBP-1, IGFBP-2, and IGFBP-6 are more severely higher, and IGFBP-3 is moderately lower.

## Research Design and Methods

### Human Subjects and Serum Samples

All study subjects were the participants of the PAGODA/PANDA study (20). Blood samples from these participants were collected during the subject’s visit to endocrine clinic. No dietary restrictions were imposed for participation in the study and blood collection. Presence of diabetic complications were determined by the attending physician/endocrinologist, based on eye exams, spot urinary albumin tests, and other clinical tests according to the ADA (21) and NFK KDOQI^1^ guidelines. The demographic characteristics for all study subjects are summarized in Table 1. The study was approved by the Institutional Review Board at Augusta University and written informed consent was obtained from adult subjects and legal guardians for minors, in accordance with the guidelines of the Declaration of Helsinki. Blood samples were collected in serum separator tubes (BD Biosciences) and allowed to clot for 30 min at room temperature. Aliquots of plasma and serum were prepared immediately after phlebotomy into wells of 96-well plates (150 μl/well) to create master plates. Daughter plates were then created by pipetting 5–25 μl of serum/well to avoid repeated freeze/thaw for all samples.

**Table 1:** **Characteristics of the patient population**.

Group	*n*	Female (%)	Age	T1D duration
**A. Discovery dataset**				
Controls	665	53.2	22.8 ± 18.1	NA
T1D (all)	584	53.9	29.3 ± 18.5	13.2 ± 12.2
T1D no complications	453	53.4	23.2 ± 15.2	9.5 ± 8.8
T1D any complication	131	55.7	50.3 ± 12.4	26.2 ± 13.3
T1D with 1 complication	78	55.1	49.0 ± 13.6	22.1 ± 13.7
T1D with 2 complications	28	53.6	50.2 ± 9.3	31.5 ± 8.9
T1D with ≥3 complications	25	60.0	54.6 ± 10.4	32.8 ± 11.6
**B. Confirmation dataset**				
Controls	1328	55.6	22.2 ± 18.0	NA
T1D (all)	1085	51.9	30.6 ± 18.2	15.2 ± 13.2
T1D no complications	830	49.5	24.9 ± 15.8	10.8 ± 10.5
T1D any complication	255	59.6	49.0 ± 12.6	29.2 ± 11.2
T1D with 1 complication	142	59.9	45.7 ± 13.5	26.4 ± 10.8
T1D with 2 complications	62	66.1	51.6 ± 9.1	31.9 ± 11.5
T1D with ≥3 complications	51	51.0	54.8 ± 10.6	33.8 ± 9.7

### Study Design

To minimize false-positive results, we employed a two-stage study design that includes a large discovery sample set and a larger confirmation sample set. The discovery dataset included 665 healthy auto-antibody negative controls and 584 T1D patients, whereas the confirmation set included a total of 1328 controls and 1085 T1D patients. The clinical and demographic information on the discovery and the confirmation datasets are presented in Table 1.

### Luminex Assays

Serum protein levels were measured using Luminex bead array kits from Millipore (Millipore Inc., Billerica MA, USA) according to manufacturer’s protocol. The kit is based on sandwich immuno-assay, which consists of dyed microspheres conjugated with a specific monoclonal capture antibody. Briefly, serum samples were incubated with capture antibodies immobilized on polystyrene beads for 1 h. The beads were then washed and further incubated with biotinylated detection antibody cocktail for 1 h. Beads were washed twice to remove unbound detection antibody and then incubated with phycoerythrin-labeled streptavidin for 30 min. Finally, beads were washed and suspended in 60 μl of wash buffer. The median fluorescence intensities (MFI) were measured on a FlexMAP 3D array reader (Millipore, Billerica, MA, USA) using the following instrument settings: events/bead: 50, minimum events: 0, flow rate: 60 μl/min, sample size: 50 μl, and discriminator gate: 8000–13500. Before profiling, the serum dilutions were optimized, by performing the assays at different serum dilutions to ensure that the majority of the data falls within the linear range of the standard curve.

### Statistical Analyses

All statistical analyses were performed using the R language and environment for statistical computing (R version 2.15.1; R Foundation for Statistical Computing^2^). The comparisons between group means were made by ANOVA (for ≥3 groups) followed by pair-wise comparisons using Bonferroni *post hoc* testing. The statistical significance of differences was set at *p* < 0.05. The effect of age on the serum levels of each candidate molecule was determined using a linear regression of protein concentration with age as covariate on data stratified by sex and disease status. To examine the relationships between disease status and the serum protein levels, logistic regression was used by including age and sex as covariates. To assess the odds ratios of having T1D or complications at different levels of each protein, subjects were divided into five quintiles based on protein levels. The cutoff protein levels for these quintiles were then used to count controls and cases in each quintile. The first quintile was used as reference, and odds ratios of having disease was calculated for upper four quintiles using Pearson’s chi-squared test with Yates’ continuity correction. The chi-squared test for trend in proportions was used to calculate the *p*-value of overall trend. Risk scores (equal to odds ratio) were assigned to each subject based on individual protein levels. For a combination of proteins, the combined risk score of each subject was calculated by simply adding risk score from multiple proteins. The odds ratios of having disease were calculated for upper four groups against the reference group as mentioned above.

## Results

Serum levels of five IGFBPs were measured in T1D patients and healthy controls to discover the alterations associated with T1D and/or its complications. To minimize false-positive results, a total of 3662 serum samples were divided into a discovery phase (1249 samples) and a confirmation phase (2413 samples). Clinical characteristics of the patient population in both sets are presented in Table 1. In the discovery phase, five IGFBP proteins (IGFBP-1, -2, -3, -6, and -7) were measured using a smaller sample set [665 antibody negative controls (AbN) controls; 584 T1D subjects]. Significant changes were observed in the levels of IGFBP-1, -2, -3, and -6, therefore, these four proteins were moved forward to the confirmation phase using a larger cohort of samples (1328 AbN controls; 1085 T1D subjects).

### IGF-Binding Proteins Are Significantly Altered in T1D and Its Complications

The status of diabetic complications in T1D subjects was captured from the medical charts, and the subjects with and without complications were examined separately. The protein levels were examined by separating the T1D patients into four groups (T1D without complications and T1D with 1, 2, or ≥3 complications). Comparison of the mean circulating levels between T1D patients without complications and controls revealed significant differences for two proteins in both discovery and confirmation sets (IGBP-1: 1.25- and 1.34-fold; IGFBP-2: 1.40- and 1.92-fold). Moderate changes (3–14%) were observed in the levels of IGFBP-3, IGFBP-6, and IGFBP-7 (Table 2).

**Table 2:** **Serum concentration of IGF-binding proteins in ABN controls and T1D patients**.

Protein	ABN (*n* = 665)	T1D_NoC (*n* = 453)	1Comp (*n* = 78)	2Comp (*n* = 28)	3Comp (*n* = 25)
**A. Discovery dataset**					
IGFBP1	867 (350–2175)	1090 (554–2237)	1361 (649–3690)	2226 (1309–4813)	2272.4 (1590–3376)
(pg/ml)		1.25 (2.4 × 10^−3^)	1.57 (2.0 × 10^−3^)	2.55 (7.0 × 10^−4^)	2.62 (1.4 × 10^−7^)
IGFBP2	4.66 (1.92–11.29)	6.50 (2.83–16.8)	13.6 (5.8–28.2)	25.8 (12.0–56.7)	30.1 (11.5–52.4)
(ng/ml)		1.40 (5.6 × 10^−4^)	2.91 (1.4 × 10^−9^)	5.53 (9.6 × 10^−7^)	6.48 (1.5 × 10^−9^)
IGFBP3	15.8 (12.2–20.6)	15.2 (11.6–20.4)	12.1.(9.4–156)	12.5 (8.9–18.5)	13.5 (10.1–20.3.)
(μg/ml)		0.97 (0.24)	0.77 (1.4 × 10^−6^)	0.79 (0.031)	0.86 (0.15)
IGFBP6	61.8 (45.5–84.4)	67.2 (50.1–92.0)	95.7 (75.5–122.3)	111.4 (75.3–143.7)	146 (104–206)
(ng/ml)		1.09 (2.6 × 10^−3^)	1.55 (1.3 × 10^−14^)	1.80 (7.5 × 10^−6^)	2.36 (1.5 × 10^−9^)
IGFBP7	86.8 (71.1 – 108.4)	78.3 (63.53–99.8)	87.43 (67.3–108.7)	91.8 (69.8–113.4)	109.1 (75.1–139.4)
(ng/ml)		0.91 (2.6 × 10^−5^)	1.01 (0.87)	1.06 (0.56)	1.26 (0.023)

**Protein**	**ABN (*n* = 1328)**	**T1D_NoC (*n* = 830)**	**1Comp (*n* = 142)**	**2Comp (*n* = 62)**	**3Comp (*n* = 51)**

**B. Confirmation dataset**					
IGFBP1	861 (315–2699)	1152 (528–2913)	1418 (604–2911)	1618 (1024–3325)	2120 (1069–4709)
(pg/ml)		1.34 (4.6 × 10^−7^)	1.65 (7.7 × 10^−7^)	1.88 (2.7 × 10^−5^)	2.45 (2.6 × 10^−7^)
IGFBP2	4.2 (1.65–10.0)	8.06 (3.62–17.6)	16.1 (7.80–36.4)	23.4 (9.21–57.0)	28.3 (12.7–66.7)
(ng/ml)		1.92 (1.2 × 10^−34^)	3.84 (2.6 × 10^−27^)	5.59 (7.4 × 10^−15^)	6.75 (1.5 × 10^−14^)
IGFBP3	14.4 (12.9–18.5)	13.5 (11.9–17.4)	10.9 (10.4–15.0)	12.8 (11.1–15.6)	11.4 (8.8–15.3)
(μg/ml)		0.94 (0.031)	0.76 (2.6 × 10^−4^)	0.89 (0.054)	0.79 (8.1 × 10^−4^)
IGFBP6	60.6 (44.6–86.9)	68.6 (52.0–96.2)	94.4 (74.3–128.4)	94.4 (68.0–139.4)	131.6 (86.8–193.4)
(ng/ml)		1.14 (1.2 × 10^−7^)	1.56 (1.5 × 10^−15^)	1.56 (1.9 × 10^−6^)	2.17 (2.2 × 10^−13^)

*First row: mean (25th–75th percentile); second row: fold change vs. ABN (*p*-value)*.

*ABN, antibody negative controls; T1D_NoC, T1D, no complications; 1Comp, T1D any complication; 2Comp, T1D with 2 complications; 3Comp, T1D with ≥3 complications*.

Comparison of the mean circulating levels between T1D patients with complications and controls revealed greater changes in IGFBP-2 (3–6-fold), IGFBP-1 (1.5–2.5-fold), and IGFBP-6 (1.5–2.5-fold) in both discovery and confirmation sample sets, whereas the levels of IGFBP-3 were moderately but significantly reduced (~0.8-fold). Furthermore, serum levels of IGFBP-1, -2, and -6 were increased with increase in number of complications (Table 2).

### Influence of Covariates on Serum Protein Levels

We next examined the potential influence of various covariates on serum protein levels in controls and T1D patients with or without complications. Significant correlations with age and sex were observed. Therefore, logistic regression analyses were carried out using protein concentration after adjusting for age and sex. In these regression analyses, IGFBP-1, -2, -6, and -7 showed moderate but significant associations with T1D (Table 3). Whereas, in T1D with complications IGFBP-1, -2, -3, and -6 showed highly significant associations (IGFBP-1: OR = 2.4, 4.1, 8.2; IGFBP-2: OR = 2.4, 4.8, 8.4; IGFBP-3: OR = 0.48, 0.52, 0.67; IGFBP-6: OR = 1.8, 3.6, 10, for 1Comp, 2Comp and 3Comp groups, respectively) (Table 3). These results suggest that the association between T1D complications and these serum proteins cannot be accounted for by the covariates examined in this study. These findings could be confirmed using an independent confirmation dataset.

**Table 3:** **Logistic Regression Analysis using protein concentration, before and after adjusting for covariates**.

Test		Unadjusted		Adjusted	
	Protein	OR	95% CI	OR	95% CI
**A. Discovery dataset**					
T1D_NoC vs. ABN	IGFBP1	1.196	(1.060–1.350)	1.221	(1.077–1.385)
	IGFBP2	1.296	(1.119–1.505)	1.280	(1.104–1.489)
	IGFBP3	0.929	(0.823–1.049)	0.929	(0.821–1.049)
	IGFBP6	1.220	(1.071–1.392)	1.264	(1.089–1.470)
	IGFBP7	0.762	(0.670–0.864)	0.754	(0.661–0.857)
1Comp vs. ABN	IGFBP1	1.359	(1.094–1.69)	2.443	(1.801–3.372)
	IGFBP2	2.474	(1.832–3.41)	2.365	(1.632–3.520)
	IGFBP3	0.568	(0.449–0.714)	0.478	(0.357–0.636)
	IGFBP6	2.940	(2.215–3.971)	1.838	(1.285–2.676)
	IGFBP7	1.024	(0.802–1.319)	0.794	(0.590–1.069)
2Comp vs. ABN	IGFBP1	1.826	(1.303–2.576)	4.095	(2.499–7.167)
	IGFBP2	3.936	(2.498–6.506)	4.757	(2.641–9.522)
	IGFBP3	0.627	(0.450–0.889)	0.523	(0.339–0.811)
	IGFBP6	3.588	(2.360–5.702)	3.561	(2.016–6.776)
	IGFBP7	1.183	(0.796–1.803)	1.011	(0.630–1.668)
3Comp vs. ABN	IGFBP1	1.885	(1.314–2.722)	8.153	(3.895–20.107)
	IGFBP2	5.013	(3.003–8.975)	8.421	(3.728–23.227)
	IGFBP3	0.724	(0.508–1.065)	0.668	(0.412–1.071)
	IGFBP6	7.460	(4.392–13.915)	10.06	(4.595–26.378)
	IGFBP7	2.047	(1.316–3.20)	1.833	(1.064–3.323)
**B. Confirmation dataset**					
T1D_NoC vs. ABN	IGFBP1	1.237	(1.134–1.351)	1.305	(1.193–1.43)
	IGFBP2	1.801	(1.632–1.993)	1.782	(1.614–1.972)
	IGFBP3	0.905	(0.825–0.990)	0.915	(0.834–1.001)
	IGFBP6	1.289	(1.171–1.421)	1.222	(1.1–1.36)
1Comp vs. ABN	IGFBP1	1.427	(1.198–1.712)	2.283	(1.824–2.895)
	IGFBP2	3.151	(2.568–3.906)	3.444	(2.716–4.432)
	IGFBP3	0.780	(0.690–0.883)	0.713	(0.616–0.835)
	IGFBP6	2.881	(2.293–3.654)	1.725	(1.324–2.283)
2Comp vs. ABN	IGFBP1	1.578	(1.215–2.087)	2.942	(2.053–4.362)
	IGFBP2	4.250	(3.136–5.905)	4.364	(3.05–6.49)
	IGFBP3	0.875	(0.738–1.109)	0.751	(0.581–1.075)
	IGFBP6	2.883	(2.079–4.055)	1.524	(1.034–2.297)
3Comp vs. ABN	IGFBP1	2.000	(1.468–2.804)	3.985	(2.607–6.408)
	IGFBP2	5.144	(3.630–7.551)	6.396	(4.044–10.844)
	IGFBP3	0.814	(0.691–1.010)	0.656	(0.504–0.928)
	IGFBP6	6.408	(4.312–9.947)	3.694	(2.3–6.159)

*ABN, antibody negative controls; T1D_NoC, T1D no complications; 1Comp, T1D any complication; 2Comp, T1D with 2 complications; 3Comp, T1D with ≥3 complications*.

### Analysis of Serum Protein Levels Using Age Matched Dataset

**Figure 1:**
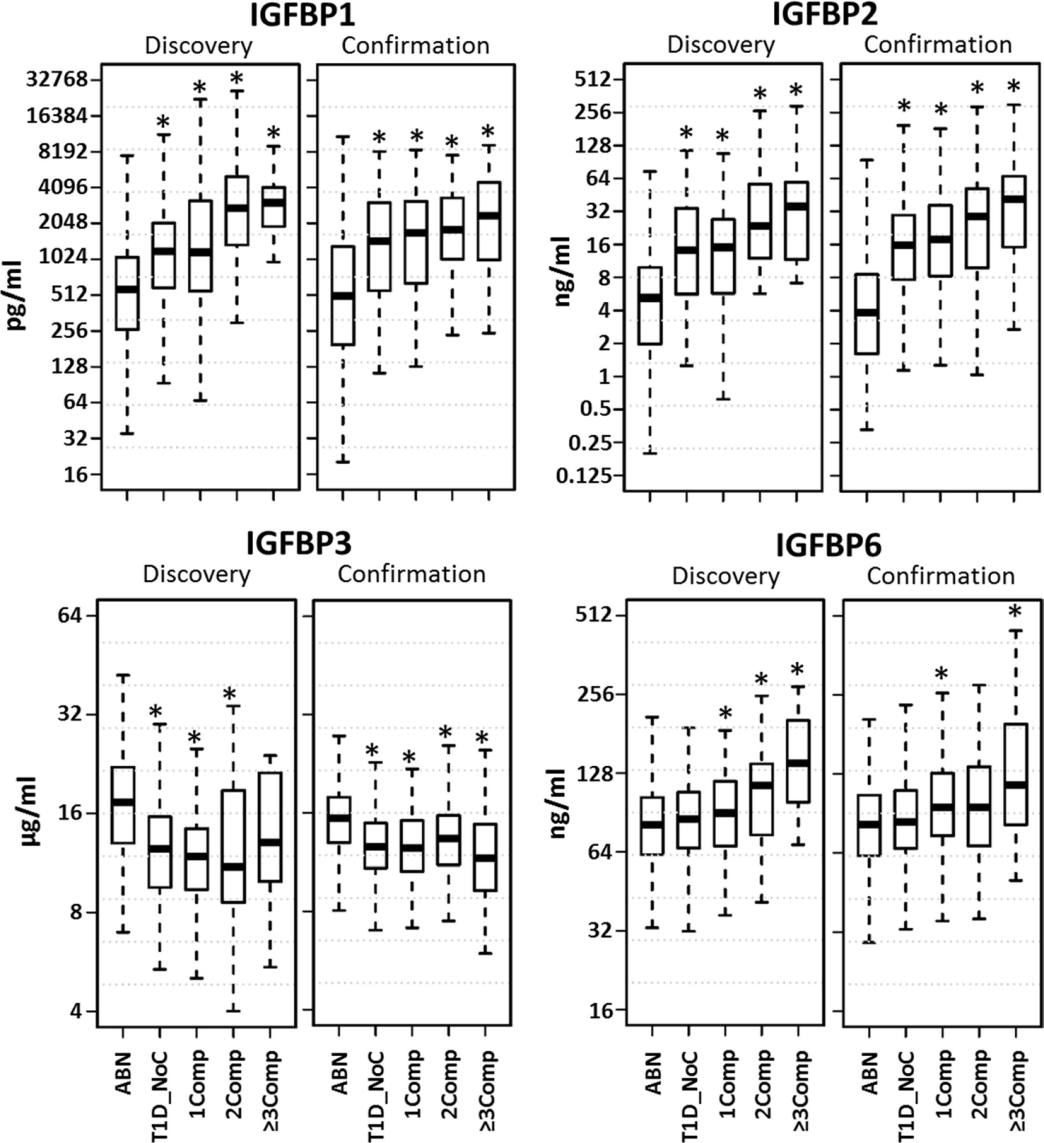
**Distribution of serum protein levels in age matched datasets**. Boxplots depict the distribution of the protein levels in five different groups (ABN, antibody negative controls; T1D_NoC, T1D no complications; 1Comp, T1D with any complication; 2Comp, T1D with 2 complications; 3Comp, T1D with ≥3 complications). A clear increasing trend was observed for IGFBP-1, IGFBP-2, and IGFBP-6 with increase in number of complications. *p < 0.05.

Since the healthy control and T1D without complication cohorts were fairly large, we decided to censor these groups to better age match with complication patients. Boxplots depicting the distribution of the protein levels in matched data are shown in Figure 1. A clear increasing trend was observed for IGFBP-1, IGFBP-2, and IGFBP-6 with increase in number of complications. Since similar findings were observed in both discovery and confirmation sets, we merged these datasets for further analyses.

### Risk of T1D and Its Complications with IGFBP Alterations

**Figure 2:**
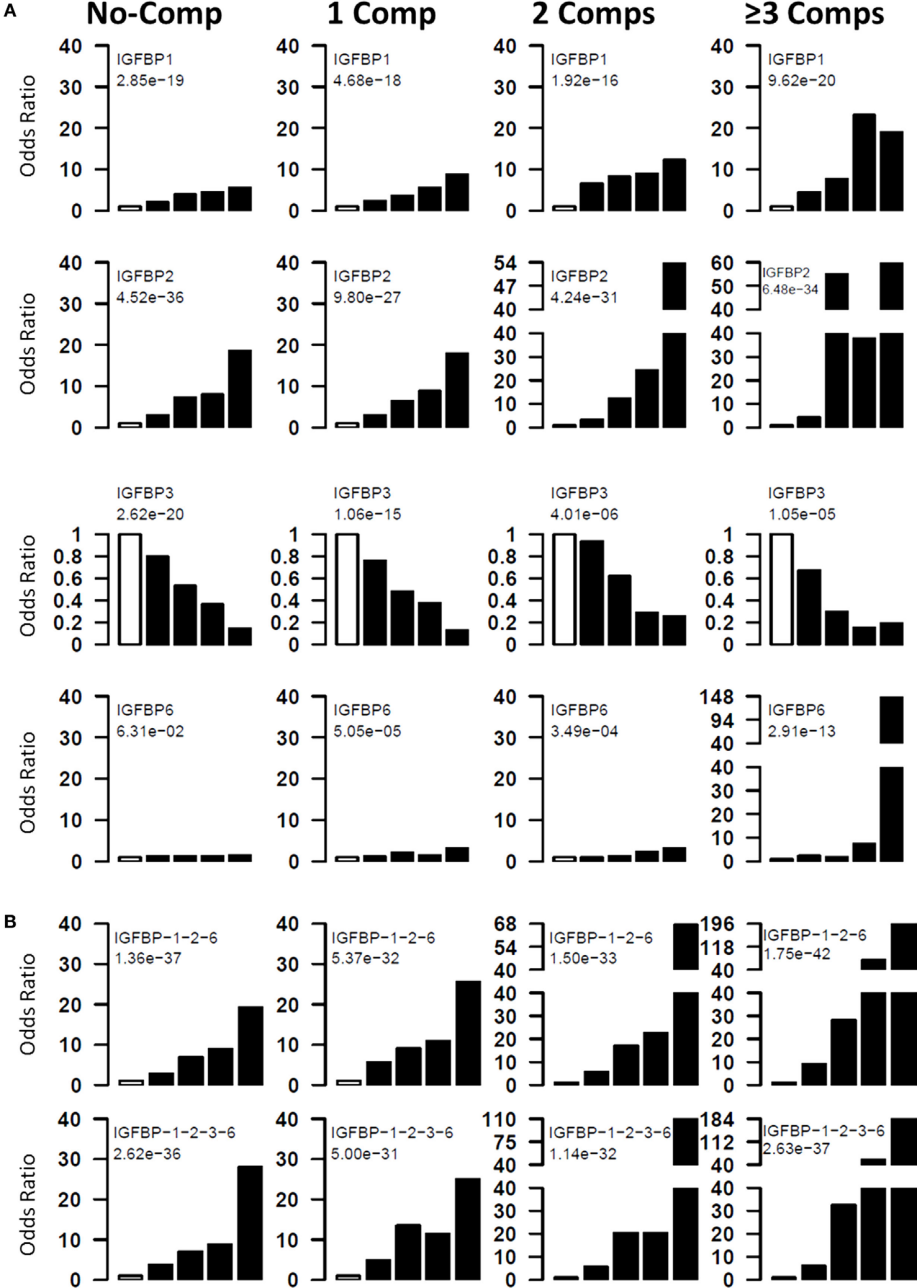
**Risk of T1D and its complications with IGFBP alterations**. Four groups (T1D_NoC, 1Comp, 2Comp, and ≥3Comp) were compared to ABN controls. Individual proteins **(A)** and protein combinations **(B)** were used to assess the odds ratios of having T1D or complications at different protein levels. All subjects were divided into five quintiles based on protein levels. The first quintile was used as reference, and odds ratios of having disease was calculated for upper four quintiles. The chi-squared test for trend in proportions was used to calculate the *p*-value of overall trend. Analyses using quintiles revealed that risk of T1D and complications increases with increasing concentrations of IGFBP-2, IGFBP-1, and IGFBP-6, whereas, IGFBP-3 has a negative association with T1D complications. The open bar represents the first quintile as reference (OR = 1). From left to right, each of the other four solid bars represent second to fifth quintiles.

The risk of having diabetic complications at different levels of serum proteins was examined by computing the odds ratios at five quintiles. Four different groups of T1D patients (0, 1, 2, ≥3 complications) were compared with healthy controls. For these analyses, cutoff values for each protein were determined by dividing the patient group into five quintiles. These cutoff values were used to assign the healthy controls and patients into five different groups. Odds ratios for the top four quintiles were computed using bottom quintile as reference (Figure 2A). For T1D-noComp, the strongest association is observed with IGFBP-2 (*p*-trend <10^−35^), which has an OR of 18.6, for the fifth quintile. IGFBP-3 has the second strongest association (*p*-trend <10^−19^) with OR of 0.18 for the top quintile. IGFBP-1 is the third best protein with maximum OR of 5.5 (*p*-trend <10^−18^).

For T1D complications, the strongest association is observed with IGFBP-2 (*p*-trend = 10^−27^–10^−35^) followed by IGFBP-1 (*p*-trend = 10^−16^–10^−20^), IGFBP-3 (*p*-trend = 10^−05^–10^−15^), and IGFBP-6 (*p*-trend = 10^−04^–10^−13^). These analyses clearly suggest that risk of having complication increases with increasing IGFBP-1, -2, and -6 and with decreasing IGFBP-3 levels (Figure 2A).

### Protein Combinations Define Patients at High Risk of Developing T1D and Complications

Since multiple IGFBPs are associated with complications, we attempted to examine the combined effect of these proteins. For this purpose, we calculated risk score of each subject by adding the quintile odds ratios from individual proteins and then examined association between disease and the risk scores. The combinations improved the highest OR values as well as the proportion of T1D subjects with the highest OR (Figure 2B). The best three protein model was IGFBP-1 + IGFBP-2 + IGFBP-6, which improved the ORs associated with the third to fifth quintile (third to fifth quintile ORs: noComp = 7–19; 1Comp = 9–25; 2Comp = 17–67; 3Comp = 28–196). Four-protein model further improved the highest OR over the three protein models (third to fifth quintile ORs: noComp = 7–28; 1Comp = 11–25; 2Comp = 20–110; 3Comp = 32–184) (Figure 2B).

## Discussion

This study was undertaken to profile IGFBPs in T1D and its complications using a large patient cohort. We found moderate changes in the levels of several IGFBPs in T1D and more severe alterations in the presence of diabetic complications. An increase in IGFBP-1, IGFBP-2, and IGFBP-6 levels along with decrease in IGFBP-3 was significantly associated with the presence of diabetic complications. These findings have potential clinical implications, as IGFBP complexes may serve as better therapeutic targets to ameliorate the decreased IGF signaling in T1D, potentially to prevent diabetic complications.

Insulin-like growth factor 1-binding protein-3 prolongs IGF half-life in the circulation and serves as the main carrier of IGF to the cell-surface. On the other hand, IGFBP-1 and IGFBP-2 have a well-known role in metabolic regulation, maintaining blood glucose levels, insulin sensitivity, glucose intolerance, and hyperinsulinemia (22–24). In addition, IGF-independent actions of IGFBPs are an emerging area of research.

Insulin-like growth factor 1-binding protein-3 is the most important carrier of IGF, which is bound to about 75% of IGF in the blood circulation and promotes somatic growth by increasing the IGF-I access to its cellular receptors. It is well established that high levels of IGFBP-3 are associated with growth stimulation (25). In our study, we found a significant association between the decreased levels of IGFBP-3 and T1D complications (second quintile OR = 0.67–0.93; third quintile OR = 0.29–0.8; fourth quintile OR = 0.15–0.53; fifth quintile OR = 0.13–0.36). IGFBP-3 has also been shown to have IGF-independent functions and acts as an anti-inflammatory molecule. In human aortic endothelial cells, IGFBP-3 inhibits TNF-α, CRP, and high glucose-induced NF-κB activity and subsequently suppresses monocyte adhesion to endothelial cells through the IGFBP-3 receptor (16). Reduced levels of IGFBP-3 in T1D complications may result in suppression of its anti-inflammatory functions, and, therefore, IGFBP-3 may present itself as a therapeutic target for events occurring during the development of complications. Also, in a recent study, it has been shown that IGFBP-3 inhibits retinal endothelial cell apoptosis through activation of an IGFBP-3 receptor in a hyperglycemic environment (26). IGFBP-3 increases endothelial NO synthase expression in human endothelial progenitor cells leading to NO generation and provides cytoprotection following vascular injury (27). Our study and accumulating evidences in recent literature confirm the role of IGFBP-3 in diabetic complications, however further studies are needed to elucidate the precise mechanisms.

Unlike IGFBP-3, the actions of other IGFBPs are very different. IGFBP-1 levels are low during the growth phase but increase several-fold during prolonged catabolic states due to growth inhibition. IGFBP-1 concentrations are dynamically regulated in response to nutritional status (13). The plasma insulin levels are inversely correlated with the IGFBP-1 levels. Inhibition of IGFBP-1 transcription by insulin is mainly conferred by insulin response element in the IGFBP-1 promoter region. In our study, we found significantly higher levels of IGFBP-1 in both T1D with and without complications as compared to controls; however, the differences were larger in T1D with complications (second quintile OR = 2–6; third quintile OR = 4–8; fourth quintile OR = 4–23; and fifth quintile OR = 5–19). Poor glycemic control in T1D is associated with elevated serum IGFBP-1 levels and reduced serum IGF-I levels, particularly where microvascular complications are present (9). Increasing age is accompanied by a further increase in IGFBP-1 levels in both T1D and T2D subjects. This age- and diabetes-dependent increase in IGFBP-1 and subsequent decrease in IGF-I activity accelerate endothelial cell death and reduce the regenerative capacity of these cells, offering a possible mechanism for the development of vascular complications (9).

In our study, the largest increase was observed in the levels of IGFBP-2 (~2-fold in T1D alone and ~4-fold in T1D with complications). Quintile analyses revealed increased risk of T1D and complications with increase in IGFBP-2 levels (second quintile OR = 3–4; third quintile OR = 6–55; fourth quintile OR = 8–38; and fifth quintile OR = 18–60). IGFBP-2, the major IGFBP expressed in infancy, has been linked to childhood obesity and is the predominant IGFBP produced from adipocytes. IGFBP-2 is known to act as an important link between nutrition, growth, and metabolism, but its association with diabetic complications remains poorly understood. A recent study demonstrates that IGFBP-2 is a predictor of longitudinal deterioration of renal function in type 2 diabetes (28). Another study in T2D patients revealed that baseline concentrations of IGFBP-1 and IGFBP-2 are associated with longitudinal elevation in HDL-cholesterol (29).

Insulin-like growth factor 1-binding protein-6 is unique among all IGFBPs because it has a preferential affinity for IGF-II. Inhibition of IGF-II actions is the major action of IGFBP-6; however, a number of studies suggest IGF-independent actions of IGFBP-6. A recent study using a non-IGF-II binding analog of IGFBP-6 has shown IGF-II-independent roles for IGFBP-6 in inhibition of cellular proliferation (30). In our study, we found increased IGFBP-6 levels in T1D complications.

Systemic administration of free IGF-I has restricted therapeutic potential due to instability in the circulation and side effects. On the other hand, IGFBPs can produce more stable IGF signaling in the vascular compartments. Studies have shown that IGF-I/coupled with IGFBP-3 affords more efficient protection from insulitis, β-cell destruction, and T1D than IGF-I and has a potential as a prophylactic therapy in the prevention of autoimmune T1D (31).

In conclusion, we found that elevated serum levels of IGFBP-1, -2 and, -6 were associated with T1D, and its complications and IGFBP-3 level was found to be decreased in T1D with complications. As IGFBPs are proving to be better therapeutic potential in metabolic disorders as compared to IGF, studies in this direction will help to define both diagnostic and therapeutic roles for IGFBPs in T1D and its complications. Also, these data provide us a foundation to explore IGF-independent mechanisms of IGFBPs during the development of diabetes and its complications.

## Author Contributions

AS and J-XS contributed to overall study design, data interpretation and manuscript preparation. AS and SP researched and analyzed the data, contributed to writing the manuscript. SS and WZ reviewed/edited manuscript. SB and SRP helped in statistical analysis. DH, LS, BB, and SA contributed to sample and clinical information collection.

## Conflict of Interest Statement

The authors declare that the research was conducted in the absence of any commercial or financial relationships that could be construed as a potential conflict of interest.

## Footnotes

^1^http://www2.kidney.org/professionals/KDOQI/guideline_diabetes

^2^www.r-project.org

## References

[1] Sorensen JS Birkebaek NH Bjerre M Pociot F Kristensen K Hoejberg AS Residual beta-cell function and the insulin-like growth factor system in Danish children and adolescents with type 1 diabetes. J Clin Endocrinol Metab (2015) 100:1053–61. 10.1210/jc.2014-3521 25532040

[2] Bideci A Camurdan MO Cinaz P Demirel F. Ghrelin, IGF-I and IGFBP-3 levels in children with type 1 diabetes mellitus. J Pediatr Endocrinol Metab (2005) 18:1433–9. 10.1515/JPEM.2005.18.12.1433 16459470

[3] Hedman CA Frystyk J Lindstrom T Chen JW Flyvbjerg A Orskov H Residual beta-cell function more than glycemic control determines abnormalities of the insulin-like growth factor system in type 1 diabetes. J Clin Endocrinol Metab (2004) 89:6305–9. 10.1210/jc.2004-0572 15579794

[4] Frystyk J Bek T Flyvbjerg A Skjaerbaek C Orskov H. The relationship between the circulating IGF system and the presence of retinopathy in Type 1 diabetic patients. Diabet Med (2003) 20:269–76. 10.1046/j.1464-5491.2003.00921.x 12675639

[5] Kim MS Lee DY. Insulin-like growth factor (IGF)-I and IGF binding proteins axis in diabetes mellitus. Ann Pediatr Endocrinol Metab (2015) 20:69–73. 10.6065/apem.2015.20.2.69 26191509 PMC4504992

[6] Ekman B Nystrom F Arnqvist HJ. Circulating IGF-I concentrations are low and not correlated to glycaemic control in adults with type 1 diabetes. Eur J Endocrinol (2000) 143:505–10. 10.1530/eje.0.1430505 11022197

[7] Higashi Y Quevedo HC Tiwari S Sukhanov S Shai SY Anwar A Interaction between insulin-like growth factor-1 and atherosclerosis and vascular aging. Front Horm Res (2014) 43:107–24. 10.1159/000360571 24943302 PMC4199335

[8] Toth P Tarantini S Ashpole NM Tucsek Z Milne GL Valcarcel-Ares NM IGF-1 deficiency impairs neurovascular coupling in mice: implications for cerebromicrovascular aging. Aging Cell (2015) 14:1034–44. 10.1111/acel.12372 26172407 PMC4693458

[9] Janssen JA Lamberts SW. Circulating IGF-I and its protective role in the pathogenesis of diabetic angiopathy. Clin Endocrinol (Oxf) (2000) 52:1–9. 10.1046/j.1365-2265.2000.00922.x 10651746

[10] Mohan S Baylink DJ. IGF-binding proteins are multifunctional and act via IGF-dependent and -independent mechanisms. J Endocrinol (2002) 175:19–31. 10.1677/joe.0.1750019 12379487

[11] Jogie-Brahim S Feldman D Oh Y. Unraveling insulin-like growth factor binding protein-3 actions in human disease. Endocr Rev (2009) 30:417–37. 10.1210/er.2008-0028 19477944 PMC2819737

[12] Firth SM Baxter RC. Cellular actions of the insulin-like growth factor binding proteins. Endocr Rev (2002) 23:824–54. 10.1210/er.2001-0033 12466191

[13] Wheatcroft SB Kearney MT. IGF-dependent and IGF-independent actions of IGF-binding protein-1 and -2: implications for metabolic homeostasis. Trends Endocrinol Metab (2009) 20:153–62. 10.1016/j.tem.2009.01.002 19349193

[14] Bach LA. Recent insights into the actions of IGFBP-6. J Cell Commun Signal (2015) 9:189–200. 10.1007/s12079-015-0288-4 25808083 PMC4458248

[15] Beattie J Hawsawi Y Alkharobi H El-Gendy R. IGFBP-2 and -5: important regulators of normal and neoplastic mammary gland physiology. J Cell Commun Signal (2015) 9:151–8. 10.1007/s12079-015-0260-3 25645979 PMC4458252

[16] Mohanraj L Kim HS Li W Cai Q Kim KE Shin HJ IGFBP-3 inhibits cytokine-induced insulin resistance and early manifestations of atherosclerosis. PLoS One (2013) 8:e55084. 10.1371/journal.pone.0055084 23383064 PMC3557269

[17] Hjortebjerg R Tarnow L Jorsal A Parving HH Rossing P Bjerre M IGFBP-4 fragments as markers of cardiovascular mortality in type 1 diabetes patients with and without nephropathy. J Clin Endocrinol Metab (2015) 100:3032–40. 10.1210/jc.2015-2196 26046968

[18] Bereket A Lang CH Wilson TA. Alterations in the growth hormone-insulin-like growth factor axis in insulin dependent diabetes mellitus. Horm Metab Res (1999) 31:172–81. 10.1055/s-2007-978716 10226799

[19] Peet A Hamalainen AM Kool P Ilonen J Knip M Tillmann V Circulating IGF1 and IGFBP3 in relation to the development of beta-cell autoimmunity in young children. Eur J Endocrinol (2015) 173:129–37. 10.1530/EJE-14-1078 25947142

[20] Carmichael SK Johnson SB Baughcum A North K Hopkins D Dukes MG Prospective assessment in newborns of diabetes autoimmunity (PANDA): maternal understanding of infant diabetes risk. Genet Med (2003) 5:77–83. 10.1097/01.GIM.0000055196.67008.1B 12644776

[21] American Diabetes A. Standards of medical care in diabetes – 2014. Diabetes Care (2014) 37(Suppl 1):S14–80. 10.2337/dc14-S014 24357209

[22] Kelley KM Oh Y Gargosky SE Gucev Z Matsumoto T Hwa V Insulin-like growth factor-binding proteins (IGFBPs) and their regulatory dynamics. Int J Biochem Cell Biol (1996) 28:619–37. 10.1016/1357-2725(96)00005-2 8673727

[23] Rajaram S Baylink DJ Mohan S Insulin-like growth factor-binding proteins in serum and other biological fluids: regulation and functions. Endocr Rev (1997) 18:801–31. 10.1210/er.18.6.801 9408744

[24] Rechler MM Insulin-like growth factor binding proteins. Vitam Horm (1993) 47:1–114. 10.1016/S0083-6729(08)60444-6 7680510

[25] Rosenfeld RG Hwa V. The growth hormone cascade and its role in mammalian growth. Horm Res (2009) 71(Suppl 2):36–40. 10.1159/000192434 19407495

[26] Zhang Q Soderland C Steinle JJ. Regulation of retinal endothelial cell apoptosis through activation of the IGFBP-3 receptor. Apoptosis (2013) 18:361–8. 10.1007/s10495-012-0793-3 23291901 PMC4026254

[27] Kielczewski JL Jarajapu YP McFarland EL Cai J Afzal A Li Calzi S Insulin-like growth factor binding protein-3 mediates vascular repair by enhancing nitric oxide generation. Circ Res (2009) 105:897–905. 10.1161/CIRCRESAHA.109.199059 19762684 PMC3635679

[28] Narayanan RP Fu B Heald AH Siddals KW Oliver RL Hudson JE IGFBP2 is a biomarker for predicting longitudinal deterioration in renal function in type 2 diabetes. Endocr Connect (2012) 1:95–102. 10.1530/EC-12-0053 23781310 PMC3681324

[29] Narayanan RP Fu B Oliver RL Siddals KW Donn R Hudson JE Insulin-like growth factor-II and insulin-like growth factor binding protein-2 prospectively predict longitudinal elevation of HDL-cholesterol in type 2 diabetes. Ann Clin Biochem (2014) 51:468–75. 10.1177/0004563213499145 24081183

[30] Raykha C Crawford J Gan BS Fu P Bach LA O’Gorman DB. IGF-II and IGFBP-6 regulate cellular contractility and proliferation in Dupuytren’s disease. Biochim Biophys Acta (2013) 1832:1511–9. 10.1016/j.bbadis.2013.04.018 23623986

[31] Chen W Salojin KV Mi QS Grattan M Meagher TC Zucker P Insulin-like growth factor (IGF)-I/IGF-binding protein-3 complex: therapeutic efficacy and mechanism of protection against type 1 diabetes. Endocrinology (2004) 145:627–38. 10.1210/en.2003-1274 14617576

